# Five Cases of Operation by the Bloodless Method*Reported to the Medical Society of the county of Albany, N. Y., April 28, 1875.

**Published:** 1875-07

**Authors:** Lewis Balch

**Affiliations:** Albany, New York


### FIVE CASES OF OPERATION BY THE BLOODLESS METHOD.*

BY LEWIS BALCH, M.D., ALBANY, NEW YORK.

Operations without loss of blood are attracting a great deal of attention at the present time. Allow me to present five cases in which Esmarch's apparatus was used.

Case I. James Dunbar, æt. 22, Ireland, painter, was admitted to St. Peter's Hospital last September, suffering from anchylosis of left ankle and necrosis of left tibia, of nine months' standing. It was decided to operate, and October 10th, the patient being in condition, he was etherized, Esmarch's apparatus applied, and on removal of the elastic bandage, a crucial incision, about 2½ by 1½ inches, was made in the lower third of the tibia. No blood followed this incision, and the dissection was easily accomplished, the involucrum exposed perforated in several places with a bone trephine, and the

*Reported to the Medical Society of the county of Albany, N. Y., April 28, 1875.

sequestrum removed. Upon the removal of the sequestrum, there was some little oozing from the granulations below it, but the sponge had to be used but once or twice. When the elastic ligature was loosed, the capillary hemorrhage was quite free, but was readily controlled by stuffing the wound with charpie soaked in a solution of carbolic acid, gr. v. to water ℥i. No vessels were secured.

Case II. Michael Duyer, æt. 10, Ireland, entered the hospital having a large indolent ulcer on one foot, the result of an injury caused by a railroad car, by which the toes were amputated a year ago. Every method having been tried to heal the ulcer, it was decided, in consultation, to remove the metatarsal bones and allow the granulations to contract. Patient being in condition, he was etherized October 21st, and Esmarch's apparatus applied, the ulcer being just covered over with lint and oiled paper to prevent soiling the bandage. On removal of the bandage, an incision was made, carried as far back as the metatarsal articulations. No blood followed, the knife and sponges were not used. The flaps thus made were turned back and the bones separated at the joint. Some oozing took place on the elastic ligature being removed, which was controlled by the same means employed in the last case. Two days after the operation, patient complained of some pain in the groin, and, on examination, the inguinal glands were found enlarged. A reddish line extended from the foot to the groin, more distinct in some portions than others, but readily traceable throughout its whole extent. The enlarged glands did not suppurate, and the cyraphatics and inflammation disappeared under hot applications.

Case III. Lucy Butler, æt. 10, Ireland, entered the hospital for disease of the os calcis, resulting from an injury received during last summer. In consultation, it was decided to operate by scooping out the inside of the bone, avoiding complete removal, if possible. Being in condition, she was etherized November 23d, and Esmarch's bandage applied in the same manner as in the foregoing case. A semi-lunar incision was made over the bone, midway between the external malleolus. The upper flap thus formed was raised with the periosteum and the bone penetrated with the trephine. No blood was seen oozing during the whole operation, until the ligature was removed, when it began to flow quite rapidly. At the suggestion of Dr. Swinburne, the foot and leg were plunged into a bucket of hot water in order to restore more quickly capillary circulation and venous return. The wound was stuffed with lint wet with carbolic acid lotion, gr. v. to water ℥i.

Case IV. John M'Laughlin, æt. 22, Ireland, employed at West Albany to couple cars, had his right hand caught between the bumpers, and entered the hospital the 4th of December. Examination showed fractures of the first and second fingers and thumb, with extensive laceration of the soft parts. Upon consultation, it being decided to operate, the patient was etherized and Esmarch's apparatus applied in the usual manner. The operation consisted of resection of the phalangeal articulation of the thumb and amputations of the first finger at the meta-carpo-phalangeal joint—the head of the meta-carpal bone being removed by the saw—and the second finger at the first phalangeal joint. No vessels were ligated, and no blood was seen during the operation. Some little oozing took place upon the removal of the elastic ligature, which was easily stopped. The wound was closed by nine silver wire sutures and lint soaked in the solution of carbolic acid, gr. v. to water ℥i., applied.

Case V. Daniel M'Dermot, æt. 58, Ireland, was admitted to St. Peter's Hospital with a compound comminuted "crush" of the right foot, caused by a locomotive. The leg was denuded of skin for the lower third. It was decided, upon consultation, to amputate. The patient was accordingly etherized. Owing to the extensive laceration, Esmarch's apparatus was not applied the whole distance from the foot up, but merely over that part of the limb still covered by the skin. At the first stroke of the knife, some blood flowed, but betore the bones were uncovered the operation had become bloodless. The anterior and posterior tibial arteries were tied before the ligatures were loosened, but the interosseous having retracted a little, some hemorrhage took place before it was fully secured.

Taking into consideration, howeve·, that the same condition of retraction would have caused the same amount of hemorrhage under any circumstances, the patient did not lose as much blood as would have been lost in the ordinary mode of controlling bleeding.

In all the cases where the operation was below the knee, the elastic ligature was applied just above that joint. The cord used was round, and, to protect the soft parts from undue pressure, two or three turns of a roller were taken around the limb before retricting.

In the operations on the hand, compression was made at the elbow, the same precaution with the roller being observed.

In all cases where the elastic bandage could be applied to the whole limb, nearly absolute freedom from blood was apparent, rendering the operation much easier to perform. Only in one instance did there seem to be any ill effect from the pressure, and that was in the case of the boy Duyer, when lymphitis ending at the injiguneal ganglea threatened quite a formidable complication, and I am inclined to think that the application of the pressure over the suppurating surface may have forced some pus corpuscles into the lymphatics. Therefore, in subsequent operations, where there was pus, I avoided drawing the bandage over the part. The amount of pressure necessary for the proper application of the bandage is of importance, but I know of no way by which any rule could be given to enable one to regulate the pressure so as to obtain the desired result, and not do injury to the vessels and nerves by using too much force. So far, in my hands, the apparatus has been entirely satisfactory, and has materially lessened the difficulties of the operations. I regret that the time restriction was kept up was not more carefully noticed, but the longest time was in the case of M'Laughlin, when it was continuous for thirty-five minutes, and no trouble followed.

